# Ockham’s razor defeated: about two atypical cases of hemolytic uremic syndrome

**DOI:** 10.1186/s12882-020-01926-2

**Published:** 2020-07-11

**Authors:** Chloe Schwarz, Alice Brehon, Cyril Mousseaux, Yosu Luque, Patricia Senet, Patricia Mariani, Inna Mohamadou, Lara Zafrani, Véronique Frémeaux-Bacchi, Eric Rondeau, David Buob, Cédric Rafat

**Affiliations:** 1 Service d’urgences néphrologiques et transplantation rénale, hôpital Tenon, Paris, France; 2 Service de dermatologie, hôpital Tenon, Paris, France; 3 Service de microbiologie, hôpital Robert Debré, Paris, France; 4 Service de Médecine Intensive et Réanimation, hôpital Saint Louis, Paris, France; 5 Service d’immunologie, Hôpital Européen Georges Pompidou, Paris, France; 6 Service d’anatomopathologie, hôpital Tenon, Paris, France

**Keywords:** HUS, STEC, Physiopathology, aHUS, Parvovirus B19, Case report

## Abstract

**Background:**

Medical investigation is a favorite application of Ockham’s razor, in virtue of which when presented with competing hypotheses, the solution with the fewest assumptions should be privileged. Hemolytic uremic syndrome (HUS) encompasses diseases with distinct pathological mechanisms, such as HUS due to shiga-like toxin-producing bacteria (STEC-HUS) and atypical HUS, linked to defects in the alternate complement pathway. Other etiologies such as Parvovirus B19 infection are exceptional. All these causes are rare to such extent that we usually consider them mutually exclusive. We report here two cases of HUS that could be traced to multiple causes.

**Cases presentation:**

Case 1 presented as vomiting and diarrhea. All biological characteristics of HUS were present. STEC was found in stool (by PCR and culture). After initial remission, a recurrence occurred and patient was started on Eculizumab. Genetic analysis revealed the heterozygous presence of a CFHR1/CFH hybrid gene. The issue was favorable under treatment.

In case 2, HUS presented as fever, vomiting and purpura of the lower limbs. Skin lesions and erythroblastopenia led to suspect Parvovirus B19 primo-infection, which was confirmed by peripheral blood and medullar PCR. Concurrently, stool culture and PCR revealed the presence of STEC. Evolution showed spontaneous recovery.

**Conclusions:**

Both cases defy Ockham’s razor in the sense that multiple causes could be traced to a single outcome; furthermore, they invite us to reflect on the physiopathology of HUS as they question the classical distinction between STEC-HUS and atypical HUS. We propose a two-hit mechanism model leading to HUS. Indeed, in case 1, HUS unfolded as a result of the synergistic interaction between an infectious trigger and a genetic predisposition. In case 2 however, it is the simultaneous occurrence of two infectious triggers that led to HUS. In dissent from Ockham’s razor, an exceptional disease such as HUS may stem from the sequential occurrence or co-occurrence of several rare conditions.

## Background

Scientific investigation is a favorite application of William Ockham’s *lex parsimonia* by virtue of which “*Entia non sunt multiplicanda praeter necessitatem”* or in other words “More things should not be used than are necessary*”.* Although there are numerous formulations of this principle, a widely accepted clinical corollary is that when contemplating multiple competing hypotheses, the one with the fewest assumptions is to be privileged. Hemolytic uremic syndrome (HUS) is a rare and complex clinical syndrome defined by thrombocytopenia, non-immune microangiopathic hemolytic anemia, and acute kidney injury [1]. Shiga-like toxin-producing bacteria (STEC) represents the most common form of HUS, yet it remains a rare occurrence with an estimated incidence in the European Union of 1.7 cases per 10^5^ patient.years (http://ecdc.europa.eu/en/publications/Publications/food-and-waterborne-diseases-surveillance-report-2015.pdf). In comparison, atypical hemolytic uremic syndrome (aHUS), a complement mediated disease, is even more infrequent with a reported estimated incidence of 0.23 per year per 10^6^ people in the French population [2]. In fact, the spectrum of HUS encompasses a myriad of other etiologies, many being exceptional and merely supported by a handful of case reports and a plausible biological rationale. Such is the case of parvovirus B19, a virus with a distinct tropism for the endothelium which has been acknowledged as a rare cause of HUS [3–5]. In agreement with Ockham’s razor, it may be posited that the likelihood of a case of HUS having more than one cause is very poor and, correlatively, that the confident identification of one cause may obviate the need for further etiological investigation. Herein we describe two cases of HUS that defy Ockham’s razor to the extent that both cases could be traced to multiple causes.

## Cases presentation

### Case 1

**Fig. 1:**
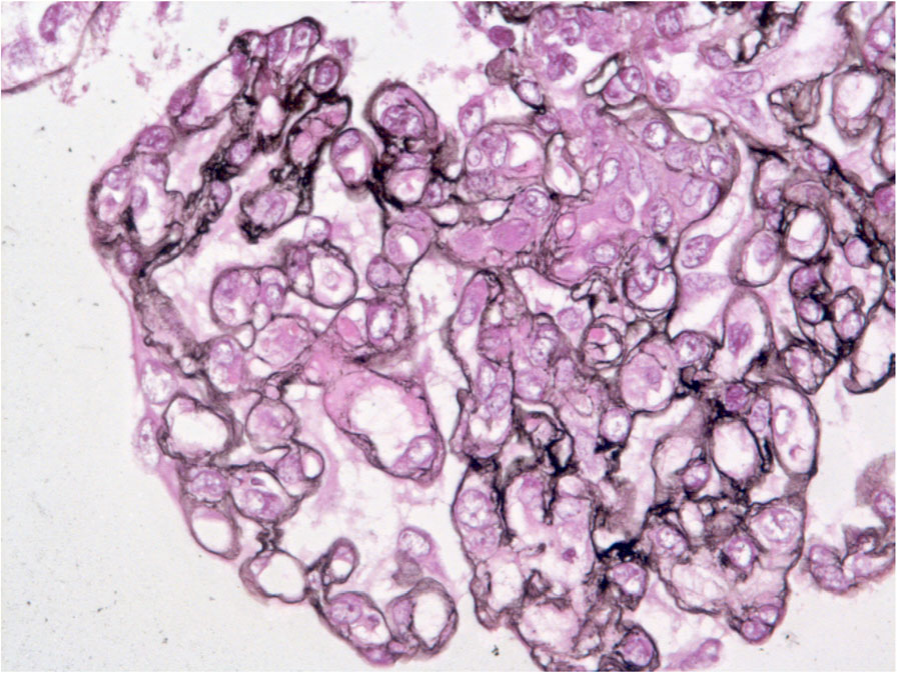
Renal biopsy of Patient 1 showed glomerular thrombotic microangiopathy with reduplication of basement membranes and prominent endothelial swelling (silver stain)

A 34-year-old male patient with an unremarkable medical history consulted his local emergency department for intractable emesis for the past 48 h. The day before, he had been given a diagnosis of hand, mouth and feet syndrome with a possible cross-transmission from his 4-year-old daughter. He had a single bout of non-bloody diarrhea. Notable clinical signs consisted in elevated blood pressure (167/98 mmHg), purpura of the lower limbs and papulovesicular acrodermatitis of both hands and wrists. Prominent biological abnormalities consisted of KDIGO stage 3 AKI (Blood urea: 30 mmol/L plasma creatinine: 497 μmol/L) and hematological thrombotic microangiopathy (TMA) (hemoglobin levels: 13.1 g/dL, platelets: 47000 / mm^3^, haptoglobin levels < 0.20 g/dl, greatly elevated LDH levels: 1843 UI/L and schistocytes were detected). Proteinuria was in the nephrotic range (6 g/24 h), consisted primarily of albumin (66%) and was associated with microscopic hematuria upon urine culture. The patient was referred to an intensive care department and was started immediately on plasma exchange (PE). Neurological examination was normal. After two rounds, PE were discontinued owing to normal ADAMTS13 activity (113%). Additional etiological workup included negative HIV, HBV, HCV testing, unremarkable lymphocyte immunophenotyping and normal levels of C3, C4 and CH50. He was next referred to our renal intensive care unit. Quantitative explorations of the alternate complement pathway (ACP) disclosed normal levels of MCP, Factor H and Factor I and negative testing for anti-FH antibodies. Accordingly, renal biopsy identified double contours and several foci of mesangiolysis as well as focal segmental glomerulosclerosis of the tip variant affecting one glomerulus (Fig. 1). Following supportive care with rehydration and blood pressure control, signs of peripheral TMA resolved while plasma creatinine levels declined to 350 μmol/L. The presence of shigatoxin (*stx2b*) and hemolysin (*ehxa*) were detected via PCR assessment in the stool samples and stool culture confirmed the presence of the enterohemorrhagic O128 *Escherichia coli* clone. Together these results ruled in favor of STEC-HUS. One month later the patient was readmitted in our unit owing to the recurrence of peripheral thrombotic microangiopathy (platelets: 130000/mm3, Hemoglobin: haptoglobin < 0.01 g/dl, schistocytes: 1%, LDH: 740 UI/L) and AKI with a creatinine level of 470 μmol/L. Repeated stool culture did not find evidence for the persistence of the predetermined *E. coli* clone or shigatoxin. Due to gradual worsening of the kidney function and platelet levels the patient was empirically started on eculizumab pending genetic findings. In the course of 15 days, creatinine levels gradually decreased from 630 μmol/L to 193 μmol/L and signs of TMA resolved. Genetic testing revealed that the patient was a heterozygous carrier of a CFHR1/CFH hybrid gene demonstrated to be associated with aHUS [6]. An attempt to discontinue eculizumab therapy 9 months after its initiation resulted in a second recurrence with an increase of creatinine levels to 430 μmol/L along with overt signs of peripheral TMA. After resuming eculizumab therapy, TMA resolved once again and creatinine levels fell back to baseline levels.

### Case 2

**Fig. 2:**
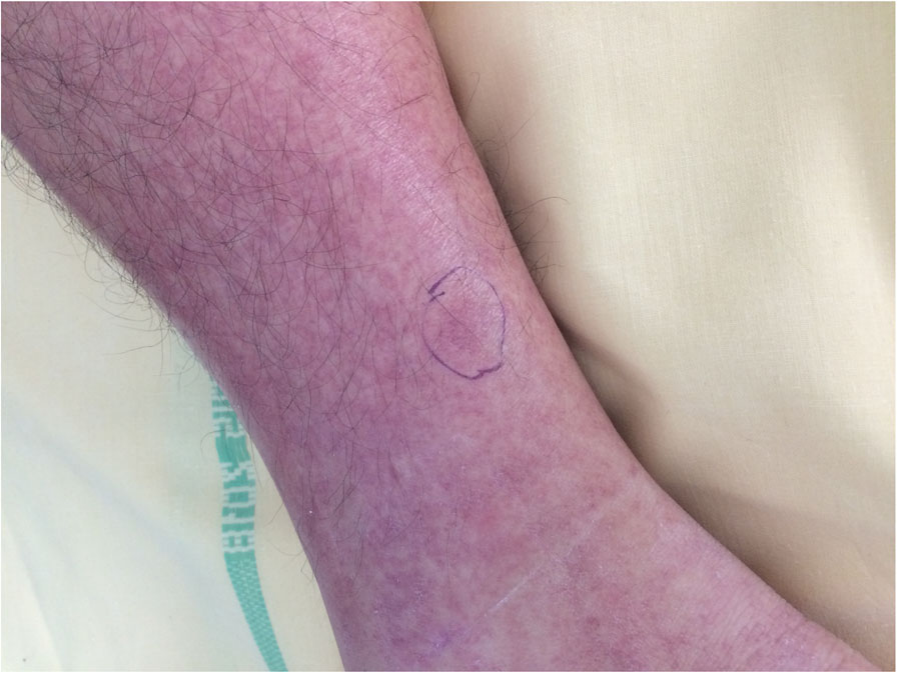
Patient 2 presented with a palpable purpura of legs

A 44-year-old male patient presented to the emergency department with fever and skin lesions. His only past medical history consisted of IV drug abuse, discontinued for several years and under substitution therapy by buprenorphin. He reported over the past 2 weeks fever, emesis, myalgia, ankle arthralgia, headache and asthenia. Clinical examination noted a purpura located on the feet and distal legs (Fig. 2). Blood pressure was 120/86 mmHg. First blood tests only revealed mild thrombocytopenia (120,000/mm3) and creatinine levels were normal (98 μmol/L). He was referred to our nephrological department 4 days later due to the subsequent development of a rapidly progressive acute kidney injury. Upon admission he presented KDIGO stage 3 AKI (serum creatinine: 404 μmol/L, BUN: 43 mmol/L) with microscopic hematuria and glomerular range proteinuria (2 g/24 h) and signs of hematological TMA (Platelets 71,000 /mm3, hemoglobin level: 10 g/dL, haptoglobin < 0.08 g/L, LDH 864 U/L, detection of schistocytes 1.7%, reticulocytes 12,000/mm3). Blood pressure remained normal, as well as neurological examination. No diarrhea was reported. Platelet levels spontaneously normalized within 24 h. A renal biopsy was performed, showing marked glomerular endotheliosis without evidence of microthrombi; no cellular proliferation was noted.

Other biological investigations included negative testing for HIV, HBV, and HCV, mildly decreased levels of C3 (0.8 g/L) and C4 (0.19 g/L) serum complement levels, and negative ANCA. Owing to the aregenerative character of anemia, a bone marrow exam was performed; it showed erythroblastopenia and no other cytological abnormality. A skin biopsy was also realized and revealed purpuric capillaritis without vasculitis. Parvovirus B19 was detected both in the plasma (6.07 log) and in the bone marrow biopsy. Parvovirus B19 serology disclosed positive anti-B19 IgM (34 UI/L) and IgG antibodies (23 UI/L).

Taken together the clinical and biological presentation was suggestive of Parvovirus B19 primo-infection. However, stool sample culture found the presence of enterohemorragic O26 *Escherichia coli* confirmed by PCR which was positive for *stx1b* (shigatoxin gene), *eae* (adhesion gene) and *ehxa* (hemolysin gene). The patient spontaneously and rapidly improved with normalization of platelets within 7 days. One month later, patient had clinically fully recovered, hemoglobin and platelets levels were normal, serum creatinine was 58 μmol/L with no significant proteinuria; isolated microscopic hematuria persisted.

## Discussion and conclusion

Both cases of HUS presented here, and summarized in Table 1, could be traced to more than one etiology. HUS is a rare entity for which the classical nosological definition accepts on one side typical or STEC-HUS, which is preceded by bloody diarrhea and is a one-time disease, and on the other side aHUS, linked to defects in the regulation of the ACP, which is typically prone to recurrences in the absence of treatment. The distinction between these two entities is purportedly based on different clinical presentations but ultimately the final diagnosis relies on isolation of shiga-like toxin producing *E.coli* strain on the one hand, and identification of genetically based or acquired complement pathway alteration on the other. Of note, pathogenic variants of the complement system and anti-CFH auto-antibodies only account for 60 to 65% of the total cases of aHUS. DGKE pathogenic variants are reported in an extra 3% of the cases which leaves more than 30% of aHUS cases undocumented based on current genetic testing [2, 7]. Pending conclusive physiopathology-based investigations, both conditions appear to overlap to the point where they are indiscernible from a bedside standpoint. In lieu of this classical separation in two distinct entities, we argue that in case 1 HUS unfolded as a two-hit mechanism, driving the activation of the ACP and the formation of microthrombi, STEC acting here as one of the most potent among different possible triggers to this condition. Indeed, next to aHUS, STEC-HUS has been shown to involve the alternate pathway complement [8]. Stx2 has been shown in vitro to interfere with ACP regulation by binding factor H [9]. Furthermore Stx2 induces the expression of P-selectin on human microvascular endothelial cell surface, which binds and activates C3 via the alternate pathway, leading to thrombi formation in a murine model of STEC-HUS [10].

**Table 1:** Summary of clinical and biological characteristics of Cases 1 and 2

	Patient 1	Patient 2
Clinical presentation	No fever Elevated blood pressure Vomiting and diarrhea Skin purpura	Fever Normal blood pressure Vomiting, no diarrhea Skin purpura Arthralgia and myalgia
Biological presentation	KDIGO stage 3 AKI Proteinuria 6 g/24 h Microscopic haematuria Hemoglobin 13.1 g/dL Platelets 47,000/mm3 Schistocytes 1% LDH 740 UI/L	KDIGO stage 3 AKI Proteinuria 2 g/24 h Microscopic haematuria Hemoglobin 10 g/dL Platelets 71,000/mm3 Schistocytes 1.7% LDH 864 UI/L
Complement exploration	Normal C3 Normal C4 Normal Factor H, Factor I, MCP Absence of anti-Factor H antibodies	Moderately low C3 Normal C4 Normal Factor H, Factor I, MCP Absence of anti-Factor H antibodies
Microbiological evidence of STEC	Stool culture positive for *E. coli* O128 serotype PCR positive for *stx2b* and *ehxa* genes	Stool culture positive for E. coli O26 serotype PCR positive for *stx1a*, *eae* and *ehxa* genes
Other cause of HUS	Presence of a heterozygous CFHR1/CFH hybrid gene	Parvovirus B19 primo-infection
Recurrence	Yes	No

This two-hit mechanism model is grounded in the concept of a synergic interaction between an infectious trigger and a predisposing genetic background. Accordingly, two cases of STEC-HUS have been reported in a 4-year old child and an 18-month infant, both shown to carry MCP mutations. The outcome proved fatal in the former case while eculizumab resulted in protracted remission in the latter [11, 12]. Similarly, Alberti et al. reported two additional cases of STEC-HUS resulting in ESRD, yet, post-transplantation recurrence of HUS revealed a mutation of CFI and MCP respectively [13].

However, a recent series by Frémeaux-Bacchi et al addressed this issue in the pediatric setting [14]: if it did not yield a higher rate of pathological variants related to ACP genes in the STEC-HUS population than in the general population (17% vs 14%), it did unravel a significantly higher frequency of very rare pathogenic variants in the STEC-HUS cohort (4% vs 0.8%).

Another model for this two-hit mechanism is the occurrence of two concurrent infectious triggers, which is deemed to be the likely scenario in case 2. Parvovirus B19 has been implicated in various renal manifestations, namely proliferative glomerulonephritis, collapsing glomerulopathy or focal segmental glomerulosclerosis as well as TMA [15]: one occurrence ascribed to parvovirus B19 primo-infection in a healthy adult was related by Prasad et al [3]; the clinical presentation was particularly severe resulting in permanent chronic dialysis. Parvovirus B19 has also been found responsible of TMA in kidney recipient patients [4, 16]. From a physiopathological standpoint, the hypothesis that Parvovirus B19 can cause TMA is supported by its tropism for the endothelial cell: direct infection and injury is permitted by the presence on the endothelial cell’s surface of the P antigen, which acts as a receptor for the virus and is found in the kidney [15, 17]. Of note, glomerular endotheliosis, the prevailing pathological finding in case 2, is both a prominent lesion in TMA and a recognized feature of parvovirus B19 renal injury [5, 18].

Another approach to these cases is foreseen in the words of Walter Chatton: “Consider an affirmative proposition, which, when it is verified, is verified only for things; if three things do not suffice for verifying it, one has to posit a fourth, and so on in turn [for four things, or five, etc.]”. (*Reportatio* I, 10–48, paragraph 57, p. 237). In case 1 STEC-HUS could not account for the relapse of TMA which occurred several weeks after the initial event. Neither could STEC-HUS be held responsible for the skin manifestations in case 2. On a more general note, a novel concept has emerged from the ever-expanding list of pathogenic variants affecting the complement or other pathways whereby HUS stems from the interaction between a trigger (infectious, immunological or toxic) and a predisposing genetic background. Herein, STEC-HUS assumed a dual role as the most powerful trigger foreseeable and a direct cause of HUS per se. In case 2, it may be surmised that HUS resulted from the combined aggression of parvovirus B19 and STEC. In each case, these reports provide ground for a model of HUS physiopathology involving a two-hit mechanism. At any rate, they highlight the need for clinicians to entertain the prospect for an additional cause of HUS whenever the clinical picture or the course appears at odds with the initial etiological diagnosis of TMA.

## Data Availability

Data sharing is not applicable to this article as no datasets were generated or analysed during the current study.

## Abbreviations

ACP: Alternate complement pathway
aHUS: Atypical hemolytic uremic syndrome
AKI: Acute kidney injury
ESRD: End-stage renal disease
HBV: Hepatitis B virus
HCV: Hepatitis C virus
HIV: Human immunodeficiency virus
HUS: Hemolytic uremic syndrome
LDH: Lactate dehydrogenase
PE: Plasma exchange
STEC: Shiga-like toxin-producing bacteria
STEC-HUS: Hemolytic uremic syndrome caused by Shiga-like toxin-producing bacteria
TMA: Thrombotic microangiopathy
